# Evaluation of Humoral Immunity to SARS-CoV-2: Diagnostic Value of a New Multiplex Addressable Laser Bead Immunoassay

**DOI:** 10.3389/fmicb.2020.603931

**Published:** 2020-11-26

**Authors:** Laurent Drouot, Sébastien Hantz, Fabienne Jouen, Aurélie Velay, Bouchra Lamia, Benoit Veber, Jean Sibilia, Marlène Lotellier, Sophie Candon, Sophie Alain, Samira Fafi-Kremer, Olivier Boyer

**Affiliations:** ^1^Normandie University, UNIROUEN, INSERM, U1234, Rouen, France; ^2^Limoges University Hospital, National Reference Center for Herpesviruses, Limoges, France; ^3^Rouen University Hospital, Department of Immunology, Rouen, France; ^4^Strasbourg University Hospital, Institute of Virology, Strasbourg, France; ^5^Pulmonology Department, Le Havre Hospital, Montivilliers, France; ^6^Department of Anesthesiology and Critical Care, Rouen University Hospital, Rouen, France; ^7^Department of Rheumatology, Strasbourg University Hospital, Strasbourg, France

**Keywords:** multiplex, luminex, COVID-19, SARS-CoV-2, ALBIA

## Abstract

Despite efforts to develop anti–severe acute respiratory syndrome coronavirus 2 (SARS-CoV-2) antibody (Ab) immunoassays, reliable serological methods are still needed. We developed a multiplex addressable laser bead immunoassay (ALBIA) to detect and quantify anti-Spike S1 and nucleocapsid N Abs. Recombinant S1 and N proteins were bound to fluorescent beads (ALBIA-IgG-S1/N). Abs were revealed using class-specific anti-human Ig Abs. The performances of the test were analyzed on 575 serum samples including 192 from SARS-CoV-2 polymerase chain reaction–confirmed patients, 13 from seasonal coronaviruses, 70 from different inflammatory/autoimmune diseases, and 300 from healthy donors. Anti-S1 IgM were detected by monoplex ALBIA-IgM-S1. Comparison with chemiluminescent assays or enzyme-linked immunosorbent assays was performed using commercial tests. Multiplex ALBIA-IgG-S1/N was effective in detecting and quantifying anti–SARS-CoV-2 IgG Abs. Two weeks after first symptoms, sensitivity and specificity were 97.7 and 98.0% (anti-S1), and 100 and 98.7% (anti-N), respectively. Agreement with commercial tests was good to excellent, with a higher sensitivity of ALBIA. ALBIA-IgG-S1/N was positive in 53% of patients up to day 7, and in 75% between days 7 and 13. For ALBIA-IgM-S1, sensitivity and specificity were 74.4 and 98.7%, respectively. Patients in intensive care units had higher IgG Ab levels (Mann–Whitney test, *p* < 0.05). ALBIA provides a robust method for exploring humoral immunity to SARS-CoV-2. Serology should be performed after 2 weeks following first symptoms, when all COVID-19 (coronavirus disease 2019) patients had at least one anti-S1 or anti-N IgG Ab, illustrating the interest of a multiplex test.

## Introduction

The emergence of the severe acute respiratory syndrome coronavirus 2 (SARS-CoV-2), which caused the coronavirus disease 2019 (COVID-19) pandemic in December 2019, has led to the development of diagnostic molecular and then serological tests. The reference standard of molecular test for diagnosis of COVID-19 is reverse transcription–polymerase chain reaction (RT-PCR), which detects viral RNA using nasopharyngeal swabs or other upper respiratory tract specimens. Therefore, RT-PCR remains the primary method of diagnosing SARS-CoV-2 despite limitations including false-negative or false-positive results due to the technique itself, insufficient amount of material at the site of sample collection, or inappropriate time of sampling. Serological tests are essential complements to molecular tests because they can identify individuals with SARS-CoV-2 at a distance from infection, when RT-PCR has become negative or was inconclusive. Besides diagnosis, serological tests are useful for epidemiological purposes, vaccination research, and, possibly, for assessment of the level of protection toward reinfection. Serological assays evaluate the humoral immune response to nucleocapsid (N) or Spike (S) proteins as they have been shown to be the most immunogenic proteins among coronaviruses (Meyer et al., 2014).

Serological tests include lateral flow immunoassays (LFIAs), chemiluminescent assays (CLIAs), bead-based assays, immunometric luminescence, electrochemiluminescence immunoassays, or enzyme-linked immunosorbent assays (ELISA). Tests typically detect the presence of antibodies (Abs) against the S protein or its domains (S1, S2, or RBD) and/or the N protein. The sensitivity of SARS-CoV-2 immunoassays may vary widely according to the time when serum samples were collected, with a higher sensitivity for CLIAs and ELISAs than for LFIAs, whereas the specificity of the different tests is typically higher than 95% (Lisboa Bastos et al., 2020).

Here, we developed a multiplex addressable laser bead immunoassay (ALBIA) to detect and quantify IgG Abs against the Spike S1 domain and nucleocapsid N, and a monoplex ALBIA to assay for anti-S1 IgM Abs.

## Materials and Methods

### Serum Samples

This is a retrospective study of serum samples from biorepositories of three French university hospitals authorized by the French Ministry of Research for the collection, analysis, storage, and reuse: Rouen University Hospital (authorization AC 2008-87), Limoges University Hospital (CRBioLim, authorization DC 2008-604), and Strasbourg University Hospital (authorization DC 2010-2222). All 192 sera analyzed, collected between March 23 and April 30, were from hospitalized or outpatients who had all been laboratory-confirmed positive for SARS-CoV-2 by RT-PCR of pharyngeal swab specimens. Of these 192 patients, 18 were hospitalized in the intensive care unit for a severe form of the disease.

Control sera were collected from 300 healthy blood donors (*Etablissement Français du Sang*, Lille, France), 13 patients with PCR-confirmed infections by other human coronaviruses (17 sera: HKU1, *n* = 3; OC43, *n* = 11; NL63, *n* = 3), and 70 patients with different inflammatory/autoimmune diseases according to established classification criteria: American College of Rheumatology revised criteria for systemic lupus erythematosus (SLE) (Tan et al., 1982) with anti-dsDNA aAbs (*n* = 12), American Rheumatism Association criteria for rheumatoid arthritis (RA) (Arnett et al., 1988) with anti-CCP Abs and/or rheumatoid factor (*n* = 23), revised European criteria for primary Sjögren syndrome (SS) (Vitali et al., 2002) with anti-SSA and/or anti-SSB aAbs (*n* = 14), and Troyanov criteria for antisynthetase syndrome (ASS) (Troyanov et al., 2005) (*n* = 21).

All serum samples were stored at −80°C until use. Handling of serum samples was performed in a BSL-2 laboratory.

### Recombinant Proteins

Polyhistidine tagged recombinant Spike subunit 1 (S1, reference 40591-V08H) and nucleocapsid protein (N, reference 40588-V08B) were obtained from Sino Biologicals (Beijing, China). The identity and purity of these recombinant proteins were first determined by 4 to 10% gradient sodium dodecyl sulfate polyacrylamide gel electrophoresis (SDS-PAGE) under non-reducing conditions, followed by Coomassie blue staining. Western blot analysis was further performed by transfer of proteins separated by non-reducing SDS-PAGE to a nitrocellulose membrane followed by incubation with anti–6 × histidine monoclonal Ab (Sigma, St. Louis, MO, United States) and revelation with corresponding secondary Ab coupled to Alexa Fluor 680 (Invitrogen, Cergy Pontoise, France) (Supplementary Figure 1).

### Multiplex Addressable Laser Bead Immunoassay (ALBIA) for the Simultaneous Detection and Quantification of Anti-S1 and Anti-N IgG in COVID-19 Patients (ALBIA-IgG-S1/N)

To simultaneously detect anti-S1 and anti-N IgG from a single sample, we used two types of beads with a specific spectral signature. Color codes of S1- and N-coupled beads were numbered 26 and 55 (Bio-Rad, Hercules, CA, United States), respectively; 10 μg of recombinant proteins was coupled to 1.25 × 10^6^ fluorescent Bio-Plex^*R*^ COOH-microspheres (Bio-Rad) with the Bio-Plex^*R*^ amine coupling kit (Bio-Rad) according to manufacturer’s protocol. After coupling, coated beads were either used immediately or stored at −20°C in the dark. Efficacy of coupling was validated using a commercial Ab recognizing the polyhistidine tag (Sigma), followed by a biotinylated goat anti-mouse IgG (Southern Biotech, Birmingham, AL, United States) secondary Ab. Revelation was then performed by incubation with 50 μL of streptavidin-R-PE (Qiagen, Venlo, Netherlands) for 10 min.

Immediately prior to their use, coated beads were vigorously agitated for 30 s. Then, a 10 μL volume of S1 and N protein-coated beads (containing 1,250 beads) was added to 100 μL of serum from patients or controls [diluted in Dulbecco phosphate-buffered saline (DPBS) plus 1% fetal bovine serum] in Bio-Plex Pro Flat bottom plates (Bio-Rad). Plates were incubated for 1 h at room temperature in the dark on a plate shaker at 650 rpm. Blank (no serum, secondary Ab only), negative controls (anti-S1 and anti-N Ab negative serum), and positive controls (human anti-S1 and anti-N Ab highly positive serum) were included in every assay. Beads were collected with a magnetic washer (Bio-Rad) and washed twice with 150 μL DPBS containing 0.1% Tween-20. Biotinylated mouse anti-human IgG-specific secondary Ab (Southern Biotech) was added at 1:2,000 dilution and incubated for 30 min at room temperature under shaking. After washing, beads were incubated with 50 μL of streptavidin-R-phycoerythrin at 1:1,000 dilution for 10 min. Finally, beads were resuspended in 100 μL of DPBS and mean fluorescence intensity (MFI) was determined on a Bio-Plex^*R*^ apparatus using the Bio-Plex^*R*^ Manager Software 4.0 (Bio-Rad) by experienced investigators (L.D., M.L.). A calibrator (i.e., a human serum from a PCR-positive COVID-19 patient) with an MFI value reaching the plateau was included in each experiment.

Serum samples were initially assayed at 1:100 screening dilution. The calibrator was used at a dilution of 1:D’ in the assay, and its level was arbitrarily set to 100 arbitrary units (AU)/mL. The Ab levels were determined at a dilution of 1:D, calculated using the following formula: ([MFI serum/MFI calibrator] × level of calibrator) x D/D’. When the MFI of a given serum sample at 1:100 dilution was higher than 70% of the calibrator MFI, further dilutions were performed. The first dilution yielding an MFI inferior to 70% of the calibrator MFI was retained for calculation of Ab titers (expressed in AU/mL).

For determination of repeatability, ALBIA was performed 30 times on the same positive serum. Coefficient of variation (CV) of the titer was determined as the ratio of the standard deviation (SD) to the mean.

Receiver operating characteristic (ROC) curves were computed by varying the threshold of positivity of the test, including one value consisting in the mean + 3 SD of negative controls.

### ALBIA for the Detection and Quantification of Anti-S1 IgM Abs (ALBIA-IgM-S1)

To detect anti-S1 IgM Abs, we used the same protocol as for ALBIA-IgG-S1/N except for the following modifications. Only S1-coupled beads were used. Anti-S1 IgM Abs were revealed using a biotinylated mouse anti-human IgM Ab (Southern Biotech) at 1:2,000 dilution for 30 min. Repeatability and Ab level were determined as described above.

### SARS-CoV-2 Ab Commercial Assays

Sera were tested using an N-based CLIA detecting IgG (Abbott SARS-CoV-2 IgG for Alinity automate), a Spike S1- and S2-based CLIA detecting IgG (Diasorin IgG for Liaison automate), and an S1-RBD–based anti–SARS-CoV-2 ELISA detecting total human Ig (Wantai SARS-CoV-2 Ab ELISA on SQ2 open platform), as per manufacturer’s instructions.

### Statistical Analysis

Statistics were performed with Prism software (GraphPad, La Jolla, CA). Ab titers were compared using the non-parametric Mann–Whitney test. Concordance between the methods was analyzed using the *K* test. The interpretation of the *K* test depends on the calculated value of the coefficient *K*: discrepancy between the two tests (*K* < 0); very low agreement (0 < *K* < 0.2); low agreement (0.2 < *K* < 0.4); moderate agreement (0.4 < *K* < 0.6); good concordance (0.6 < *K* < 0.8); and excellent agreement (0.8 < *K* < 1).

## Results

### Validation of ALBIA-IgG-S1/N and ALBIA-IgM-S1

To allow quantitative analysis of anti-S1/N IgG or anti-S1 IgM in patients, we developed two ALBIAs (ALBIA-IgG-S1/N and ALBIA-IgM-S1, respectively). For this, we used as antigen polyhistidine-tagged recombinant Spike subunit 1 (S1) and nucleocapsid protein (N) of SARS-CoV-2. The identity and purity of these proteins were confirmed by Coomassie blue staining after SDS-PAGE, revealing a unique band (Supplementary Figure 1A) that was specifically recognized by an anti–polyhistidine Ab in Western blot (Supplementary Figure 1B).

**FIGURE 1:**
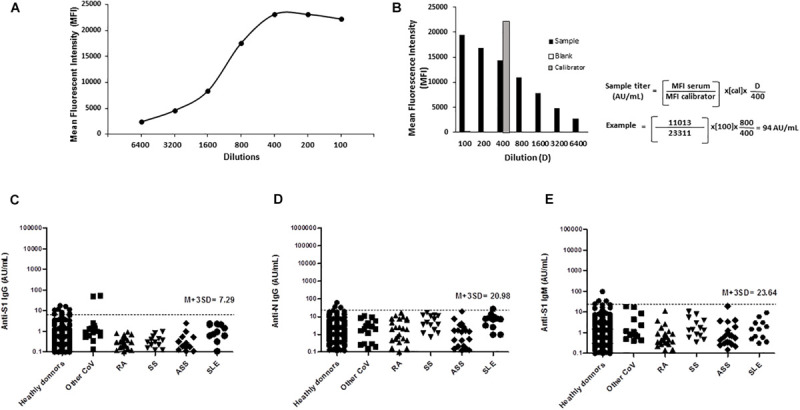
Detection, titration, and cross-reactivity of anti–SARS-CoV-2 Spike S1, nucleocapsid N protein IgG, and anti–SARS-CoV-2 Spike S1 IgM antibodies by ALBIA-IgG-S1/N and ALBIA-IgM-S1. **(A)** A calibration curve was obtained after serial dilutions of the calibrator, i.e., one highly positive sample. A plateau of MFI was reached for dilutions 1:400 or lower. **(B)** Calculation of antibody titer by reference to the MFI value of the calibrator (gray bar) used at a 1:400 dilution in the assay and its level arbitrarily set to 100 arbitrary units (AU)/mL. The assay was first performed using a 1:100 screening dilution of the serum. In case the sample’s MFI at 1/100 dilution was higher than 70% of the calibrator’s MFI, further dilutions were performed, and the first dilution yielding an MFI inferior to 70% of calibrator MFI was retained for calculation. An example is given: at 1:100 dilution, the MFI was higher than 70% of the calibrator’s MFI (23,311 × 0.7 = 16,318), requiring a 1/800 dilution for computing the titer, i.e., 94 AU/mL anti-S1 IgG level. Specificity toward non–COVID-19 patients: **(C)** anti-Spike S1 and **(D)** anti-N IgG, IgM, and **(E)** anti-Spike S1 IgM antibody reactivity in patients with different conditions: PCR-confirmed infection with other CoV (17 sera from 13 patients; HKU1, *n* = 3; OC43, *n* = 11; NL63, *n* = 3). RA, rheumatoid arthritis; SS, Sjögren syndrome; ASS, antisynthetase syndrome; SLE, systemic lupus erythematosus.

S1 and N antigens were covalently coupled to fluorescent beads and used to determine the levels of anti-S1 and N IgG Abs, or anti-S1 IgM Abs. An example of the method used for calculating anti-S1 level is illustrated in Figure 1. A calibration curve was obtained after serial dilutions of a highly anti–S1-positive serum used as calibrator. A plateau of MFI was reached for dilution 1:400 (Figure 1A). At the screening dilution of 1:100, the sample used in this example showed a saturating signal (Figure 1B). A higher 1:800 dilution was retained to compute Ab level by reference to the calibrator whose level was arbitrarily set to 100 AU/mL. The same method of calculation was used for computing the levels of anti-N IgG and anti-S1 IgM Ab.

ALBIA-IgG-S1/N was used to simultaneously investigate the presence of anti-S1 and anti-N IgG Ab. A threshold of positivity was calculated as the mean titer + 3 SD of the 300 negative control sera, which yielded values of 7.29 and 20.98 AU/mL for anti-S1 and anti-N IgG Ab, respectively (Figures 1C,D). For ALBIA-IgM-S1, this threshold was 23.64 AU/mL (Figure 1E).

To evaluate potential cross-reactivity in our ALBIA between anti–SARS-CoV-2 Ab and other human coronaviruses, we tested 17 sera from 13 patients infected with HKU1, OC43, or NL63. An IgG reactivity to S1 but not N was found only once, in two sera from the same patient sampled at two different times post-infection with human coronavirus NL63 (Figures 1C–E). In addition, 70 patients with different inflammatory/autoimmune conditions leading to the production of rheumatoid factor or other auto-Abs, e.g., SLE, RA, SS, or ASS, were further tested. They all scored negative except for one lupus patient weakly positive for anti-N IgG (Figures 1C–E).

**FIGURE 2:**
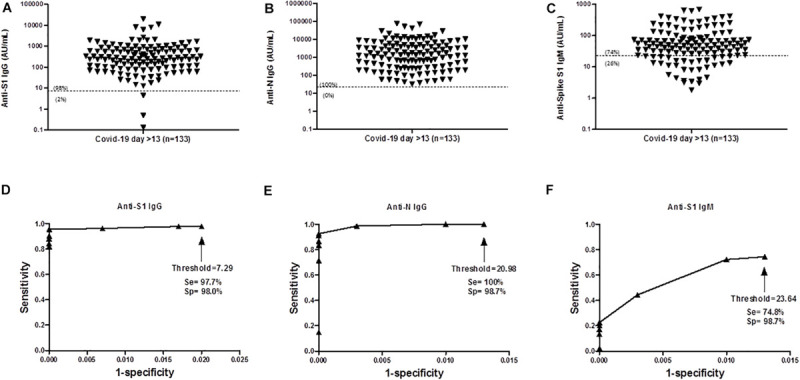
Antibody response to SARS-CoV-2 at day > 13 post infection. **(A)** Anti-S1 IgG (median = 276 AU/mL), **(B)** Anti-N IgG (median = 1,434 AU/mL), **(C)** Anti-S1 IgM level (median = 48 AU/mL). Numbers in parenthesis indicate the percentages of data above and below the threshold. **(D–F)** Receiver Operating Characteristic (ROC) curve of ALBIA-IgG-S1, ALBIA-IgG-N and ALBIA-S1-IgM. The dotted line indicates the threshold value of ‘mean + 3 standard deviations (M + 3SD)’ of the control distribution. D, day post-symptoms. Se: Sensitivity and Sp: specificity.

The diagnostic performance of the assay was determined using a collection of 133 sera from SARS-CoV-2–specific PCR-positive patients that were collected at least 14 days after first COVID-19 symptoms. ROC curve analysis of ALBIA-IgG-S1/N confirmed the accuracy of the aforementioned threshold value, i.e., mean + 3 SD. Indeed, sensitivity was 97.7% and specificity was 98.0% at a 7.29 AU/mL threshold for anti-S1 IgG (Figures 2A,D). For anti-N IgG Ab, sensitivity was 100% and specificity was 98.7% at a threshold of 20.98 AU/mL (Figures 2B,E). For ALBIA-IgM-S1, sensitivity and specificity were 74.8 and 98.7% at a threshold of 23.64 AU/mL (Figures 2C,F).

## Repeatability of Measures

Repeatability of the test was determined by calculating intra-assay variation for a given serum. CVs were 4.5 and 5.5 and 4.6% for anti-S1, anti-N IgG, and anti-S1 IgM, respectively (Supplementary Figures 2A–C), indicating a good repeatability of this ALBIA.

### Frequency of Seropositivity During the Period of Seroconversion

**FIGURE 3:**
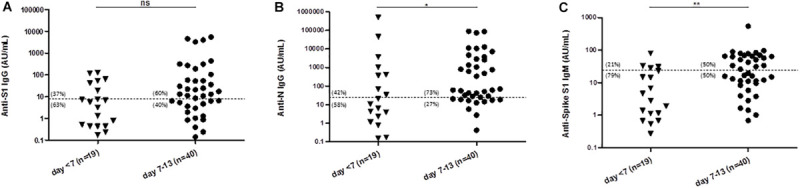
Levels of antibodies against SARS-CoV-2 at different times after symptom onset. **(A)** Level of anti-S1 IgG (median = 6 AU/mL and 13 AU/mL for day < 7 and days 7–13, respectively). **(B)** Level of anti-N IgG (median = 11 AU/mL and 60 AU/mL for day < 7 and days 7–13, respectively). **(C)** Level of anti-S1 IgM (median = 3 AU/mL and 23 AU/mL for day < 7 and days 7–13, respectively). Numbers in parenthesis indicate the percentages of data above and below the threshold. *P < 0.05, **P < 0.01 (Mann–Whitney test).

Of the 192 samples from SARS-CoV-2 PCR–positive patients analyzed herein, 19 were collected up to day 7 after symptom onset, 40 between days 7 and 13, and 133 at day 14 or more after first symptoms. In the few asymptomatic patients of this series (*n* = 3), the time of positive SARS-CoV-2 PCR was used instead. The rate of positivity increased with time for all Abs tested (Figures 3A–C). The multiplex ALBIA-IgG-S1/N scored positive in 53% in the group day <7 (as compared to 37% for anti-S1 and 42% for anti-N IgG when considered separately; Figures 3A,B), in 75% in the group days 7–13 (as compared to 60% for anti-S1 and 73% for anti-N IgG; Figures 3A,B) and 100% in the group day > 13 (as compared to 98% for anti-S1 and 100% for anti-N IgG; Figures 2A,B).

At the group level, an increase in Ab titers was observed with time (median value in group day <7, days 7–13 and day > 13: anti-S1 IgG, 6, 13, and 276 AU/mL; anti-N IgG, 11, 60, and 1,434 AU/mL; and anti-S1 IgM, 3, 23, and 48 AU/mL, respectively). All the differences between groups day > 13 and days 7–13, and between day > 13 and day <7, were statistically significant (Figure 3 and not shown). Anti-N IgG and anti-S1 IgM levels were also significantly higher in the group days 7–13 than in the group day < 7 (*p* < 0.05 and < 0.01, respectively; Figures 3B,C), although the increase of anti-S1 IgG levels was not statistically significant (*p* = 0.08; Figure 3A).

When analyzed irrespectively of time of disease onset, 161 (84%) and 170 (89%) of the 192 patients of this series were positive for anti-S1 or anti-N IgG, respectively. Ab levels in seropositive patients were highly variable, ranging from 7.5 to 19,944 AU/mL, and from 24.74 to 491,992 AU/mL for anti-S1 or anti-N IgG, respectively. Using ALBIA-IgM-S1, 123 patients (64%) were positive, with titers ranging from 24.03 to 676 AU/mL. When combining the results of the three types of Ab (IgM, IgG S1, and N), the sensitivity reached 91%.

### Ab Levels in Patients Requiring Critical Care

Of this series, 18 patients had a severe form of disease requiring hospitalization in ICU. Anti-S1 (median = 511 AU/mL) and N (median = 2,930 AU/mL) IgG levels were significantly higher in these patients than in all other patients (anti-S1 IgG, median = 126 AU/mL; anti-N IgG, median = 696 AU/mL; *p* = 0.02 and 0.04, respectively). No statistically significant difference was found for anti-S1 IgM (not shown).

### Comparison With Commercial EIA Assays

The performance of our novel assay was compared to that of different commercial assays on 76 available serum samples (10, 20, and 70 in groups day < 7, days 7–13, and day > 13, respectively). Global concordance of the multiplex ALBIA-IgG-S1/N with Diasorin and Abbott assays was 91% and 93%, respectively, with *K* coefficients of 0.64 and 0.73 indicating a good concordance. Discordant tests were as follows: positivity of ALBIA when Diasorin was negative (*n* = 6/7), negativity of ALBIA when Diasorin was positive (*n* = 1/7), and positivity of ALBIA when Abbott was negative (*n* = 5/5).

In addition, we analyzed the results of ALBIA according to the antigenic reactivity (anti-S or anti-N IgG). Concordance of ALBIA anti-S IgG with Diasorin was 93% with a coefficient *K* of 0.74 (good agreement). Concordance of ALBIA anti-N IgG with Abbott was 97% with a coefficient *K* of 0.91 (excellent agreement). Concordance of ALBIA IgG + IgM with the Wantai assay (detection of total Abs) was 95% with a *K* coefficient of 0.80 (excellent agreement).

## Discussion

In this study, we report the high sensitivity and specificity of a new multiplex ALBIA for exploring the humoral immune response to SARS-CoV-2 subunit S1 (IgG and IgM) and nucleocapsid N protein (IgG). Since the emergence of COVID-19 at the end of 2019, efforts have been made to develop serological tests whose limitations have been widely outlined (Duong et al., 2020; Lai et al., 2020; Smithgall et al., 2020). Different health authorities or scientific organizations have issued recommendations on the performance that serological tests should have, i.e., a clinical specificity of at least 98% and a clinical sensitivity of 90% or more (Farnsworth and Anderson, 2020; Haute Autorité De Santé [HAS], 2020). Our multiplex ALBIA-IgG-S1/N largely meets these criteria and confirms the excellent performance of bead immunoassays in accordance with a recent report (Ayoubaa et al., 2020). Our study further shows that the sensitivity of monoplex ALBIA-IgM-S1 remains around 75%, highlighting the fact that not all COVID-19 patients produce detectable levels of IgM (Guo et al., 2020; Liu et al., 2020).

The performance of current serological tests for COVID-19 has been judged perfectible in a large meta-analysis (Lisboa Bastos et al., 2020). Differences observed in sensitivity of such tests depend on the antigenic source used for each assay. Even if Abs directed against the viral S protein of SARS-CoV-2 are expected to appear earlier than those directed against the N protein (Liu et al., 2020), it has been shown that N–specific Abs were more sensitive than S-specific Abs for detecting early infection (Burbelo et al., 2020). Thus, multiplex assays offer several advantages. Allowing the simultaneous analysis of immune responses to different antigens, they increase the sensitivity of the test. Indeed, irrespectively of time of disease onset, the sensitivity of the multiplexed anti-S1 plus anti-N IgG assay (90%) was greater than the sensitivity of anti-S1 and anti-N IgG taken separately (84 and 89%, respectively). The sensitivity increases to 91% if the results of the anti-S1 IgM assay are also taken into account. Finally, combining several antigens in the same well reduces the cost and handling time of the assay.

Quantification of anti-S1 IgM and IgG allows the study of the population dynamics of anti-S1 IgG Ab response. Our results confirm that a 2-week delay is recommended for assaying IgG Ab in SARS-CoV-2–exposed patients in accordance with the literature (Huang, 2020). Also, the IgG levels of severely ill patients who required hospitalization in intensive care unit were significantly higher than those of patients with milder disease in accordance with a recent report (Long et al., 2020).

The diagnostic performance of ALBIA is equivalent to the best ELISAs or CLIAs reported in the literature (Bischof et al., 2020; Bryan et al., 2020; Kruttgen et al., 2020; Mahase, 2020; Montesinos et al., 2020; Traugott et al., 2020). Hence, we compared our novel assay with different commercially available CLIA or ELISA assays. Globally, our multiplex assay was more sensitive than the other assays tested. The best correlation was found with the Wantai ELISA, which detects total Abs against SARS-CoV-2 S1-RBD antigen, an assay already highlighted for its excellent performance (GeurtsvanKessel et al., 2020).

In conclusion, we have developed a highly sensitive and specific serological assay for exploring humoral immunity to SARS-CoV-2. This makes ALBIA a suitable tool for COVID-19 diagnosis and monitoring, epidemiological, or vaccination studies or for investigating the role of SARS-CoV-2 in non-typical forms of the disease (Hebert et al., 2020).

## Data Availability Statement

The raw data supporting the conclusions of this article will be made available by the authors, without undue reservation.

## Ethics Statement

Patients sera were from biorepositories authorized by the French Ministry of Research for the collection, analysis, storage and reuse: Rouen University Hospital (authorization AC 2008-87), Limoges University Hospital (CRBioLim, authorization DC 2008-604) and Strasbourg University Hospital (authorization DC 2010-2222).

## Author Contributions

LD, SH, and ML performed the methodology and testing. LD, OB, and SH wrote the original draft. FJ, AV, BL, BV, JS, SC, SA, and SF-K reviewed and edited the manuscript. All authors contributed to the article and approved the submitted version.

## Conflict of Interest

LD and OB are designated as inventors for the European Patent application EP 20315157.6 filed on April 14, 2020 in the names of Inserm, Université de Rouen and CHU de Rouen and entitled “methods for detecting the presence of coronavirus-specific antibodies in a subject.” The remaining authors declare that the research was conducted in the absence of any commercial or financial relationships that could be construed as a potential conflict of interest.

## Supplementary Material

Supplementary Figure 1 Quality controls of recombinant proteins. SDS- PAGE analysis of recombinant SARS-CoV-2 proteins after **(A)** Coomassie blue staining and **(B)** Western Blot using an antibody targeting the C-terminal histidine tag.

Supplementary Figure 2 Repeatability of ALBIA. **(A)** ALBIA-IgG-S1. **(B)** ALBIA-IgG-N. **(C)** ALBIA-IgM-S1. The assay was performed 30 times on the same sample, i.e., one serum from a PCR^+^ COVID-19 patient used at a working dilution of 1:100. Horizontal bars depict mean and standard deviation.
