# Supplementary figures and images for: Evaluation of Humoral Immunity to SARS-CoV-2: Diagnostic Value of a New Multiplex Addressable Laser Bead Immunoassay
Source: Front Microbiol. 2020 Nov 26;11:603931. doi: 10.3389/fmicb.2020.603931 (PMC7726470; doi:10.3389/fmicb.2020.603931)

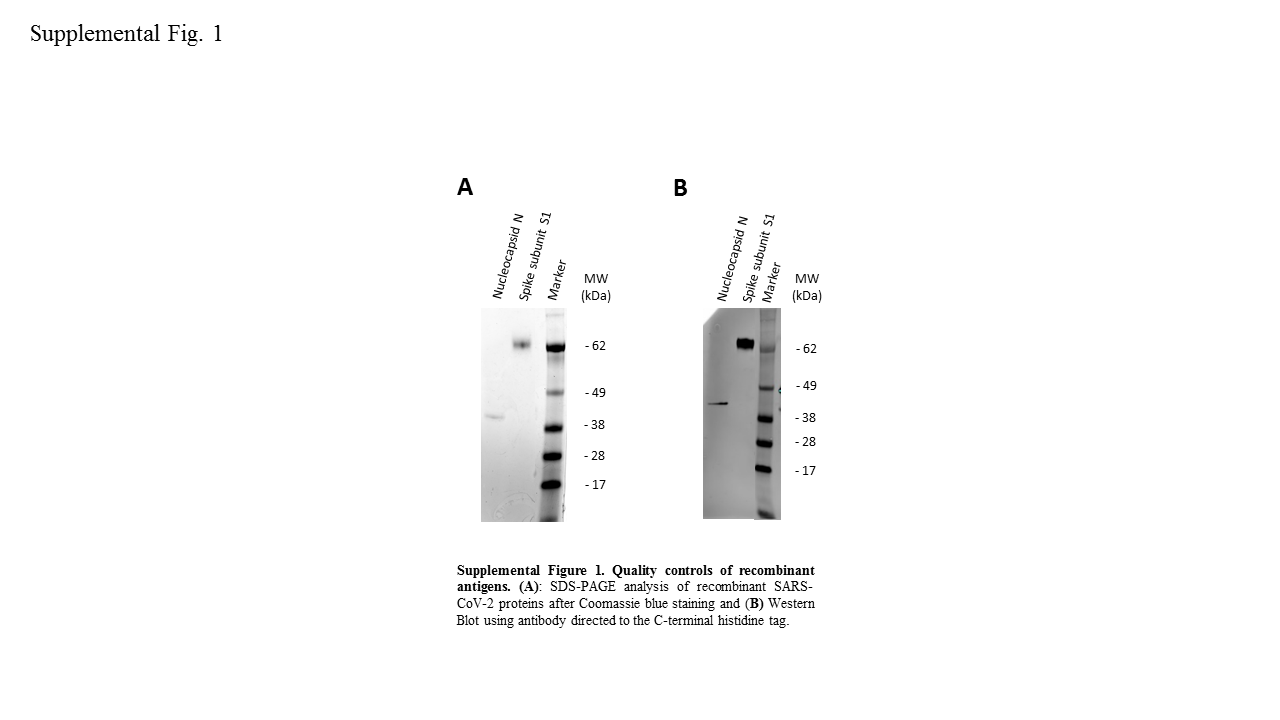

Supplement: Supplementary Figure 1 — Quality controls of recombinant proteins. SDS- PAGE analysis of recombinant SARS-CoV-2 proteins after (A) Coomassie blue staining and (B) Western Blot using an antibody targeting the C-terminal histidine tag. [file Image_1.TIF]

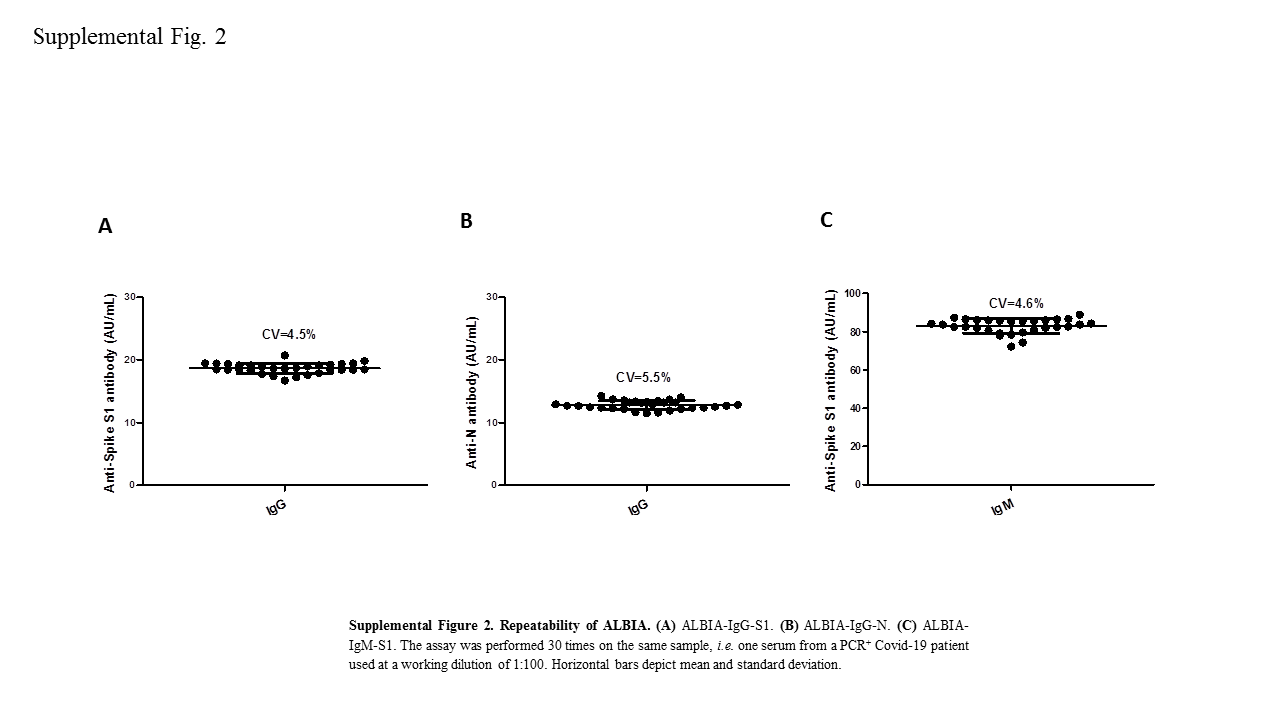

Supplement: Supplementary Figure 2 — Repeatability of ALBIA. (A) ALBIA-IgG-S1. (B) ALBIA-IgG-N. (C) ALBIA-IgM-S1. The assay was performed 30 times on the same sample, i.e., one serum from a PCR+ COVID-19 patient used at a working dilution of 1:100. Horizontal bars depict mean and standard deviation. [file Image_2.TIF]
